# Social and behavioural factors associated with risky sexual behaviours among university students in nine ASEAN countries: a multi-country cross-sectional study

**DOI:** 10.1080/17290376.2018.1503967

**Published:** 2018-07-28

**Authors:** Siyan Yi, Vannarath Te, Supa Pengpid, Karl Peltzer

**Affiliations:** a Senior Research Fellow, Saw Swee Hock School of Public Health, National University of Singapore, Singapore; b KHANA Center for Population Health Research, Phnom Penh, Cambodia; c Public Health, Touro University California, Vallejo, CA, USA; d Health Systems Research and Policy Support Unit, Ministry of Health, National Institute of Public Health, Phnom Penh, Cambodia; e ASEAN Institute for Health Development, Mahidol University, Bangkok, Thailand; f Department of Research & Innovation, University of Limpopo, Sovenga, South Africa; g HIV/AIDS/STIs/and TB (HAST), Human Sciences Research Council, Pretoria, South Africa

**Keywords:** Risky sexual behaviours, unprotected sex, multiple sexual partners, university students, ASEAN

## Abstract

While university students are potential human resources, this population group is particularly involved in health risk behaviours. Preventing risky sexual behaviours among them would contribute to prevention of HIV, sexually transmitted infections (STIs), and unwanted pregnancies, which have posed a great burden on population health. This study was therefore conducted to identify social and behavioural factors associated with risky sexual behaviours among university students in nine ASEAN countries. A multi-country, cross-sectional study was conducted in 2015 among university students by a network of researchers in the selected countries. A convenient sampling method and stratified random sampling procedures were employed to select universities and students, respectively. A structured questionnaire was translated into national languages of the participating countries and used to collect data from the selected students in the classrooms. Using STATA, Chi-square test was used to test differences in proportions, and multinomial logistic regression analyses were performed to obtain relative risk ratios and 95% confidence intervals. Multivariate logistic regression analysis was performed with to identify independent social and behavioural factors associated with non-condom use at last sexual intercourse. In total, 8,836 students with a mean age of 20.6 (SD = 2.0) participated in the study. Most of them (98.5%) were unmarried. In all countries, male students were significantly more likely to have two or more sexual partners in the past 12 months compared to female students (4.8% vs. 1.1%, *p *< 0.001). Female students were significantly more likely to report unprotected sex compared to male students (50.5% vs. 58.8%, *p *= 0.01). Results of multivariable logistic regression analyses showed that students who reported having two or more partners in the past 12 months were significantly more likely to be male, be aged between 20–30, be current tobacco smokers, be binge drinkers, have severe depressive symptoms, and have been in a physical fight in the past 12 months, compared to students who reported having less than two sexual partners in the past 12 months. Health intervention programmes to prevent and control STIs, especially HIV infection, should focus on university students having the social and behavioural characteristics that are associated with risky sexual behaviours.

## Introduction

Students in Asian universities are typically young adults in their twenties (United Nations Educational, Scientific and Cultural Organization [UNESCO], 2014). This group of population is particularly involved in risky sexual behaviours, which include, but not limited to, having sex with multiple partners, having unprotected sex (without condoms), having sexual intercourse with strangers, and having intoxicated sex (Caldeira et al., 2009). Bui et al. (2012) examined sexual behaviours of Vietnamese undergraduate female students from two universities. They found that among those who ever had boyfriends, 13% ever had vaginal sexual intercourse, and 10.3% had oral sex with their boyfriends. One-third of those having vaginal sexual intercourse did not use condoms or any contraceptive method at all during their first intercourse (Bui et al., 2012). Caldeira et al. (2009) also discovered that female college students in the United States who had vaginal sexual intercourse had intoxicated sex, unprotected sex, and sex with multiple partners in the past six months. In Thailand, 94% of university students who were moderately or highly at risk of HIV infection (18% of the total sample) did not consider themselves as being at risk or just perceived low risk, thereby tending to have unprotected sex (Khawcharoenporn, Chunloy, & Apisarnthanarak, 2015).

The risk level was classified based on pre-determined risk characteristics. For instance, those who reported having two to three sexual partners in the past 30 days would be classified into the moderate-risk group, and the high-risk group if the number of sexual partners was more than three (Khawcharoenporn et al., 2015). These similar findings were also reported among African American university students in the Midwest of the United States (Adefuye, Abiona, Balogun, & Lukobo-Durrell, 2009). Related to this low-risk perception, the African American college students did not practice safer sex for four main reasons (Duncan et al., 2002). One of them was related to negative views on the use of condoms. The other three reasons were related to feeling of invincibility, trust based on appearance or relationship quality, and desire to live for the moment. The risky behaviours and perceptions have made university students more vulnerable to sexually transmitted infections (STIs), especially HIV, and unwanted pregnancies (Bountress, Metzger, Maples-Keller, & Gilmore, 2017).

The literature has shown that there are a number of factors contributing to risky sexual behaviours among university students. It was observed that childhood abuse, poor mental health, alcohol use, drug use, partner violence, or sexual coercion are significantly correlated with risky sexual behaviours. While Peltzer, Pengpid, and Tiembre (2013) could not confirm a direct association between childhood abuses (both physical and sexual forms) and HIV risk behaviours, which included inconsistent condom use and multiple partners, Richter et al. (2014) found a strong relationship between the abuses and risky sexual behaviours among African men. Schilder et al. (2014) also found that childhood physical abuse was significantly associated with HIV incidences among young men having sex with men in Canada. In addition, Peltzer et al. (2013) revealed that poor mental health, in forms of post-traumatic stress disorder (PTSD) and depression, was associated with higher HIV risk behaviours among university students in Ivory Coast. HIV infection, in a study in South Africa, was also reported to induce PTSD and depression (Sikkema et al., 2011). Experience of childhood physical and sexual abuses, partner violence, and sexual coercion, moreover, had associations with PTSD and depression (Sikkema et al., 2011).

Risky sexual behaviours were also positively correlated with heavy alcohol consumption and drug use, e.g. marijuana use (Adefuye et al., 2009; Griffin, Umstattd, & Usdan, 2010; Kim, Celentano, & Crum, 1998; Peltzer et al., 2013; Sikkema et al., 2011). Sexual coercion, which refers to being forced to participate in a sexual situation, was found to be associated with risky sexual behaviours including multiple sexual partners, early sexual debut, and inconsistent condom use among university students in Uganda (Agardh, Odberg-Pettersson, & Östergren, 2011). The association was also true among young females aged between 10–24 years in Ethiopia (Garoma, Belachew, & Wondafrash, 2008).

In the literature, it is noticed that university students in Malaysia, Thailand, and Singapore had good understanding of HIV transmission risks (Folasayo et al., 2017; Khawcharoenporn et al., 2015; Singh, Fong, & Ratnam, 1992). However, a systematic review found that there was misunderstanding about disease transmission among college students though they were knowledgeable about the basic facts about HIV and AIDS (Lewis, Malow, & Ireland, 1997). While Duncan et al. (2002) reported that African American university students were likely to engage in risky sexual behaviours due to the four reasons mentioned above, to our knowledge, social and behavioural factors associated with risky sexual behaviours have not been well studied among university students in the Association of South-East Asian Nations (ASEAN) region.

Strengthening human resources is one of the strategic priorities in the region (ASEAN Secretariat, 2013), and preventing risky sexual behaviours among the university students would contribute to the prevention of HIV, STIs, and unwanted pregnancies (Bountress et al., 2017) and consequently contribute to the development of human resources, taking health as human capital (Bloom & Canning, 2008). STIs have posed greater burden on population health and require strong and prompt public health responses (World Health Organization [WHO], 2013). This study was therefore conducted to identify social and behavioural factors associated with risky sexual behaviours among university students in ASEAN countries. The findings would offer significant insights into planning appropriate, efficient, and effective health intervention programmes to prevent and control STIs, especially HIV infection, among university students.

## Methods

### Study design and participants

This multi-country, cross-sectional study was conducted in 2015 as part of a larger investigation on health behaviours among university students by a network of researchers in Cambodia, Indonesia, Laos, Malaysia, Myanmar, the Philippines, Singapore, Thailand, and Vietnam. A convenient sampling method was used to select universities for the survey. In Cambodia, Indonesia, and Thailand, the survey was conducted at two universities in each country. In the rest, the survey was done at one university in each country.

### Data collection and sampling procedures

A structured questionnaire was developed in English and then translated into the national languages (Bahasa, Khmer, Burmese, Laotian, Thai, and Vietnamese) of the participating countries. Back-translation into English was also performed. Well-trained research assistants administered the questionnaire in classrooms selected through a stratified random sampling procedure in which one department was randomly selected from each faculty as a primary sampling unit, and from each selected department, students were randomly selected from all the courses. The selected students self-administered the questionnaire in the classrooms. Participation rates in most of the participating countries were more than 90%, except for Indonesia (86%) and Myanmar (73%).

### Variables and measurements

#### Socio-demographic characteristics

We collected information on age, gender, residence, and family socioeconomic background. Family socioeconomic situation was assessed by asking students to rate their family background in terms of wealth as wealthy (within the highest 25% for their country), quite well-off (within the 50% to 75% range for their country), not very well-off (within the 25% to 50% range for their country), or quite poor (within the lowest 25% for their country) (Wardle & Steptoe, 1991).

#### Sexual behaviours

Participants were asked, 1) ‘During the past 12 months, with how many people have you had sex (that is, oral, anal or vaginal sex)?’ (with a response in number); 2) ‘The last time you had sexual intercourse, did you or your partner use a condom?’ (response options were ‘yes’ and ‘no’); and 3) ‘Have you ever had a disease/infection which you think you got through sexual contact?’ (response options were ‘yes’ and ‘no’) (WHO, 2017).

#### Mental health

The 10-item version of Centres for Epidemiologic Studies Depression Scale (CES-D) was used to assess depressive symptoms (Andresen, Malmgren, Carter, & Patrick, 1994). Scoring is classified from 0–9 as having a mild level of depressive symptoms, 10 to 14 as moderate depressive symptoms, and 15 representing severe depressive symptoms (Kilbourne et al., 2002). The Cronbach’s alpha reliability coefficient of this 10-item scale was 0.74 in this study.

#### Substance use

Binge drinking was measured with a question, ‘How often do you have (for men) five or more and (for women) four or more alcoholic drinks on one occasion?’ Response options were 0 = never, 1 = less than monthly, 2 = monthly, 3 = weekly, and 4 = daily or almost daily (Babor, Higgins-Biddle, Saunders, & Monteiro, 2001).

Current tobacco use was assessed with a question: Do you currently use one or more of the following tobacco products (cigarettes, snuff, chewing tobacco, cigars, etc.)? Response options were ‘yes’ or ‘no’ (WHO, 1998).

Drug use was assessed with a question, ‘How often have you taken drugs other than that prescribed by health care providers in the past 12 months?’ Response options included 1 = 0 times to 4 = 10 or more times.

## Data analyses

Data were analysed using STATA (StataCorp, LP, Texas, USA). Socio-demographic characteristics, sexual behaviours, and mental health were calculated as percentages. Chi-square test was used to test differences in proportions. Multinomial logistic regression analyses were performed to obtain relative risk ratios and 95% confidence intervals (CI) to estimate the associations between socio-demographics and mental health with having one and having two or more sexual partners in the past 12 months. Multivariable logistic regression analysis was performed with unprotected sex (non-condom use at last sexual intercourse) as the dependent variable. Independent variables significantly associated with sexual behaviours in bivariate analyses (*p *< 0.05) were included in the multivariable model. Potential multi-collinearity between variables was assessed with variance inflation factors, none of which exceeded critical value. Country was entered as the primary sampling unit for survey analyses in order to achieve accurate CIs, given the clustered nature of the data. *P* < 0.05 was considered statistically significant in all analyses.

## Ethics statement

The study was approved by an ethical committee in each country. ‘National Ethics Committee for Health Research of the Ministry of Health’, Cambodia (No. 191NECHR); ‘Ethics Committee at University of Health Sciences’, Laos; ‘Research Ethics Committee, Faculty of Medicine and Health Sciences’, Universitas Muhammadiyah Yogyakarta, Indonesia; the University of Malaya Medical Ethics Committee (MECID 201412–905), Malaysia; ‘Research and Ethical Committee of University of Medicine 1’, Myanmar; ‘Ethics Committee of the Western Visayas Health Research’, the Philippines; ‘Office of the Committee for Research Ethics (Social Sciences), the Faculty of Social Sciences and Humanities’, Mahidol University, Thailand (MU-SSIRB 2015/116(B2); and ‘Committee of Research Ethics of Hanoi School of Public Health’, Vietnam. A written or verbal consent was obtained from each participant after they were made clear that participation in the study was voluntary, and they had an opportunity to refuse or discontinue their participation at any time and for any reason. Confidentiality of the data and privacy of the participants were protected by administering the questionnaires in a private premise and excluding personal identifiers in the survey.

## Results

### Characteristics of participants

Demographic characteristics of the study sample by country are shown in Table 1. The study sample included 8,836 university students from nine ASEAN countries with a mean age of 20.6 (SD = 2.0) years. The sample size ranged from 491 in Myanmar to 1,509 in Thailand. More than half (57.1%) of them were female, and the majority (98.5%) were never married. Gender distribution varied largely within the participating countries; the proportion of female participants was only 17.7% in Thailand compared to 74.1% in the Philippines.

**Table 1.** Demographic characteristics of university students stratified by country.

Countries	Total sample	Age (years)	Female	Male	Married
	Number (%)	Mean (SD)	Number (%)	Number (%)	Number (%)
Cambodia^a^	1357 (15.4)	21.3 (2.3)	667 (49.2)	690 (50.2)	27 (2.0)
Indonesia^a^	981 (11.1)	19.3 (2.4)	695 (70.8)	286 (29.2)	32 (3.3)
Laos^a^	806 (9.1)	22.3 (1.8)	533 (66.1)	273 (33.9)	58 (7.2)
Malaysia^b^	1023 (11.6)	20.7 (1.5)	519 (50.7)	504 (49.3)	5 (0.5)
Myanmar^a^	491 (5.6)	20.1 (1.1)	282 (57.4)	209 (42.6)	1 (0.2)
Philippines^a^	958 (10.8)	18.7 (1.0)	248 (25.9)	710 (74.1)	0 (0.0)
Singapore^c^	894 (10.1)	21.2 (1.6)	443 (49.6)	451 (50.4)	4 (0.4)
Thailand^b^	1509 (17.1)	20.0 (1.3)	1242 (82.3)	267 (17.7)	1 (0.1)
Vietnam^a^	817 (9.2)	21.4 (1.6)	413 (50.6)	404 (49.4)	6 (0.7)
All	8836 (100.0)	20.6 (2.0)	5042 (57.1)	3794 (42.9)	134 (1.5)

^a^Lower middle income country.

^b^Upper middle income country.

^c^High income country.

### Sexual behaviours by country

Table 2 shows sexual behaviours among participants stratified by country. Of total, 10.8% of the participants reported having at least one sexual partner in the past 12 months. The proportion of participants who reported having one and two or more sexual partners in the past 12 months was 8.3% and 2.5%, respectively. The highest proportion of students having two or more sexual partners in the past 12 months was found in Laos (3.8%), Thailand (4.0%), and Singapore (4.3%). In all countries, male students were significantly more likely to have two or more sexual partners in the past 12 months compared to female students, with the overall rate of 4.8% and 1.1% among male and female students (*p *< 0.001), respectively. The highest rates of unprotected sex were found in Thailand (10.5%) and Laos (11.4%).

**Table 2.** Sexual behaviours in the past 12 months among university students from nine ASEAN countries.

Countries	All (*n *= 8,836)				Men (*n *= 5,042)			Women (*n* = 3,794)				Unprotected sex^1^		
	Number of sexual partner			Unprotected sex*	Number of sexual partner			Number of sexual partner						
	None	One	Two or more		None	One	Two or more	None	One	Two or more	Gender difference	Male	Female	Gender difference
	%	%	%	%	%	%	%	%	%	%	*P*-value	%	%	*P*-value
Cambodia	90.3	7.6	2.1	42.6	87.4	8.8	3.8	93.3	6.3	0.4	<0.001	45.0	27.7	0.01
Indonesia	95.4	3.9	0.7	61.3	92.0	6.3	1.7	96.8	2.9	0.3	0.002	72.2	46.2	0.14
Laos	77.5	18.6	3.8	62.3	61.2	27.5	11.4	85.9	14.1	0.0	<0.001	52.9	84.1	<0.001
Malaysia	96.6	1.8	1.7	8.0	95.4	1.8	2.8	97.7	1.7	0.6	0.02	11.8	0.0	0.31
Myanmar	95.9	1.2	2.9	48.3	91.4	2.9	5.7	99.3	0.0	0.7	<0.001	39.1	83.3	0.05
Philippines	94.5	4.5	1.0	83.0	89.0	8.5	2.5	94.6	4.5	0.9	0.02	72.7	90.3	0.09
Singapore	87.8	7.9	4.3	57.8	84.9	9.1	6.0	90.7	6.8	2.5	0.01	50.0	73.5	0.02
Thailand	79.9	16.1	4.0	58.2	59.9	29.6	10.5	84.2	13.2	2.6	<0.001	58.2	58.2	1.00
Vietnam	93.0	5.4	1.6	13.6	90.3	6.9	2.7	95.6	3.9	0.5	0.005	10.0	21.4	0.36
All	89.2	8.3	2.5	54.3	85.0	10.2	4.8	91.8	7.1	1.1	<0.001	50.5	58.8	0.01

*Only unmarried students were included in the analyses.

Among the total unmarried students, 54.2% reported that they or their partners did not use a condom at the last sexual intercourse. There was a large country variation in terms of unprotected last sex, with the highest rate found in the Philippines (83.0%), followed by Indonesia (61.3%) and Laos (62.3%). The lowest rates of unprotected sex were found in Malaysia (8.0%) and Vietnam (13.6%). Overall, female students were significantly more likely to report unprotected sex compared to male students (50.5% vs. 58.8%, *p *= 0.01). This female preponderance of unprotected last sex was true for most of the participating countries, but not in Cambodia and Indonesia, where male students were significantly more likely to report unprotected sex than female students (see Table 2).

The prevalence of STIs was remarkably low in these ASEAN countries. The proportion of students who reported having been diagnosed with an STI in the past 12 months was 0.0% in the Philippines and Vietnam, 0.1% in Malaysia, 0.2% in Thailand, 0.4% in Indonesia, Myanmar and Singapore, 0.7% in Cambodia, and 1.5% in Laos.

### Factors associated with number of sexual partners

Results of multivariable logistic regression analyses exploring risk factors associated with number of sexual partners are shown in Table 3. After adjustment for other covariates, students who reported having one sexual partner in the past 12 months were significantly more likely to be in the age groups of 20–21 (ARRR = 2.33, 95% CI = 1.15–4.74) and 22–30 (ARRR = 3.55, 95% CI = 1.64–7.68), to be binge drinkers (ARRR = 3.53, 95% CI = 1.79–6.95), and to be illicit-drug users (ARRR = 1.46, 95% CI = 1.16–1.84) compared to students who reported having no sexual partner in the past 12 months.

**Table 3.** Factors associated with sexual partnership in the past 12 months among university students from nine ASEAN countries (*n* = 8,836).

Variables	No sexual partner vs. one sexual partner		<2 vs. ≥2 sexual partners	
	CRRR (95% CI)	ARRR (95% CI)	CRRR (95% CI)	ARRR (95% CI)
*Age groups*				
18–19 (32.2%)	Reference	Reference	Reference	Reference
20–21 (49.7%)	2.75 (1.39, 5.47)**	2.33 (1.15, 4.74)*	2.84 (1.68, 4.78)**	2.49 (1.04, 5.97)*
22–30 (28.1%)	3.93 (1.44, 10.71)*	3.55 (1.64, 7.68)**	4.08 (1.58, 10.55)**	2.80 (1.23, 6.38)*
*Gender*				
Female (62.0%)	Reference	Reference	Reference	Reference
Male (38.0%)	1.54 (1.17, 2.04)**	1.17 (0.83, 1.64)	4.04 (1.72, 9.48)**	3.71 (1.39, 9.92)*
*Family economic background*				
Quite well off/wealthy (36.9%)	Reference	Reference	Reference	–
Quite poor/not very well off (63.1%)	0.83 (0.40, 1.73)	–	1.38 (0.87, 2.20)	–
*Living situation*^a^				
With parents (34.1%)	Reference	–	Reference	–
Away from parents (65.9%)	1.26 (0.47, 3.38)	–	1.38 (0.87, 2.20)	–
*Country income category*				
Upper middle income/High income (38.8%)	Reference	–	Reference	–
Lower middle income (61.2%)	0.54 (0.12, 2.40)	–	0.41 (0.14, 1.18)	–
Severe depressive symptoms (9.3%)	0.96 (0.45, 2.02)	1.13 (0.52, 2.45)	2.34 (1.23, 4.43)*	2.36 (1.33, 6.34)*
Current tobacco use (4.1%)	3.23 (1.86, 5.61)***	2.04 (0.84, 4.99)	10.80 (6.60, 17.67)***	4.57 (3.05, 6.85)***
Binge drinking (past year) (18.2%)	4.52 (2.48, 8.23)***	3.53 (1.79, 6.95)***	6.07 (3.58, 10.27)***	4.33 (2.76, 6.78)***
Illicit drug use on past year (17.4%)	1.80 (1.01, 3.23)*	1.46 (1.16, 1.84)**	1.52 (0.70, 3.28)	1.05 (0.65, 1.63)
Having been in physical fight in the past 12 months (6.9%)^a^	1.17 (0.54, 2.50)	1.01 (0.67, 1.60)	3.88 (1.87, 8.02)**	2.36 (1.37, 4.05)**

RRR = Relative Risk Ratio.

***P < .001; **P < .01; *P < .05.

^a^Not assessed in Cambodia.

Regarding multiple sexual partnership, students who reported having two or more partners in the past 12 months were significantly more likely to be in the age groups of 20–21 (ARRR = 2.49, 95% CI = 1.04–5.97) and 22–30 (ARRR = 2.80, 95% CI = 1.23–6.38), to be male (ARRR = 3.71, 95% CI = 1.39–9.92), to have severe depressive symptoms (ARRR = 2.36, 95% CI = 1.33–6.34), to be current tobacco smokers (ARRR = 4.57, 95% CI = 3.05–6.85), to be binge drinkers (ARRR = 4.33, 95% CI = 2.76–6.78), and to have been in a physical fight in the past 12 months compared to students who reported having less than two sexual partners in the past 12 months (see Table 3).

### Risk factors for unprotected sex

Table 4 shows results of multivariate logistic regression analyses exploring risk factors for unprotected sex among unmarried students. After adjustment, students who reported having unprotected sex were significantly less likely to be in the age groups of 20–21 (AOR = 0.48, 95% CI = 0.28–0.82) and 22–30 (AOR = 0.35, 95% CI = 0.19–0.57), to be male (AOR = 0.68, 95% CI = 0.43–0.88), to live away from parents (AOR = 0.37, 95% CI = 0.54), and to have two or more sexual partners in the past 12 months (AOR = 0.69, 95% CI = 0.48–0.99). Students who reported having unprotected sex were significantly more likely to report having been in a physical fight in the past 12 months (AOR = 2.56, 95% CI = 1.22–5.36).

**Table 4.** Associations with unprotected sex among unmarried university students (*n* = 8,836).

Variables	Unprotected sex	
	COR (95% CI)	AOR (95% CI)
*Age (in years)*		
18–19	Reference	Reference
20–21	0.66 (0.46, 0.96)*	0.48 (0.28, 0.82)**
22–30	0.46 (0.32, 0.68)***	0.35 (0.19, 0.57)***
*Gender*		
Female	Reference	Reference
Male	0.64 (0.50, 0.82)**	0.68 (0.45, 0.98)*
*Family economic background*		
Quite well off/wealthy	Reference	Reference
Quite poor/not very well off	0.63 (0.48, 0.81)***	0.61 (0.43, 0.88)**
*Living situation*^a^		
With parents	Reference	Reference
Away from parents	0.61 (0.45, 0.83)***	0.37 (0.26, 0.54)***
*Country income category*		
Upper middle income/High income	Reference	–
Lower middle income	1.05 (0.82, 1.35)	–
Had severe depressive symptoms	0.74 (0.50, 1.09)	–
Current used tobacco	1.18 (0.79, 1.77)	–
Had binge drinking in the past 12 months	1.25 (0.97, 1.60)	–
Used Illicit drugs in the past 12 months	1.43 (1.03, 2.00)*	1.12 (0.78, 1.64)
Been in a physical fight in the past 12 months^a^	1.61 (1.02, 2.56)*	2.56 (1.22, 5.36)*
Had ≥ 2 sexual partners in the past 12 month	0.64 (0.43, 0.86)**	0.69 (0.48, 0.99)*

COR = Crude odds ratio; AOR = Adjusted odds ratio; CI = Confidence interval.

***p < .001; **p < .01; *p < .05.

^a^Not assessed in Cambodia.

## Discussion

This study examined social and behavioural factors associated with risky sexual behaviours among university students in nine ASEAN countries. It was observed that the proportion of university students having two or more sexual partners in the past 12 months (2.5%) was much lower than the finding (19.1%) discovered by Peltzer, Pengpid, Amuleru-Marshall, Mufune, and Zeid (2016) who assessed health risk behaviours, which included sex with multiple partners in relation to religiosity, among university students from 26 countries. The risky sexual behaviours were similar in ASEAN countries included in this study irrespective of socio-economic status of the countries. Among the three countries (Singapore, Thailand, and Laos) with higher proportions of students having multiple sex partners, students in Thailand and Laos could be explained by the higher proportion of male participants who were significantly found to have sex with two or more partners in the past 12 months.

Previous studies among Asian students and at-risk young people have reported that males were significantly more likely to report having multiple sexual partners than females (Yi et al., 2010; Yi et al., 2014). Similarly, in their study in the United States, Santelli, Brener, Lowry, Bhatt, and Zabin (1998) also reported that young males aged 14–22 were more likely to have multiple sexual partners in the past three months than their female counterparts. On the contrary, in the Philippines, the majority of the participants were female; thus, the reported sex with multiple partners in the past 12 months was lower (1% of the country sample). However, the proportion of unprotected sex was quite a concern. This finding tended to support Bui et al. (2012) who revealed that female university students in Vietnam played a subordinate role and had lower self-efficacy for communicating for safer sex or asking their partners to use condoms.

In this study, the majority of the participants were unmarried. In South Africa, unmarried population, particularly those cohabitating with one another without marriage, were targeted for HIV prevention because HIV incidence among this group is the highest in the country (Shisana et al., 2016). Shisana et al. (2016) further explained that the high rate of HIV incidence among this group could be resulted from low socio-economic status, poor social cohesion, and unstable sexual relationships. It was noticed that participants in this study who had unprotected sex with multiple partners in the past 12 months were males living away from parents.

Regarding behavioural factors, findings from this study were consistent with those from other studies that found positive association between risky sexual behaviours, especially sex with multiple partners, and heavy alcohol consumption (binge drinking), drug use, and severe depression (Adefuye et al., 2009; Griffin et al., 2010; Kim et al., 1998; Peltzer et al., 2013; Sikkema et al., 2011). Moreover, this study uncovered that physical fight in the past 12 months was a factor associated with multiple sexual partnerships and unprotected sex.

This study had three main limitations. First, the main variables including sexual risk behaviours, STIs, mental health, and substance use were self-reported. In Asian cultures, these issues are sensitive and likely to be underreported. Although self-reported measures are an important source of information, considering the scarcity of data on these behaviours, especially in Asian youth, the interpretation of the findings should be made with caution. For example, it is surprising that none of the students in the Philippines and Vietnam reported having been diagnosed with an STI in the past 12 months, although it may be true that, despite having STI symptoms, they did not seek screening and/or treatment. Second, the generalisability of the study findings is questionable given that the data were collected from university students only in one or two universities in each participating country. Therefore, the extent to which these results may be generalised to students in other areas of those countries or other parts of ASEAN remains unknown. The final limitation concerns the cross-sectional nature of the study, which limited our ability to establish causality. Longitudinal, more representative studies concerning each social and behavioural factor associated with the risky sexual behaviours are necessary for future research in the region.

## Conclusions

Among university students in the nine ASEAN countries, only a small proportion of the total sample (2.5%) reported having sexual intercourse with multiple partners in the past 12 months. This risky sexual behaviour was associated with several social and behavioural factors including binge drinking, tobacco consumption, mental problems, physical fights, living away from parents, and being male. However, females were more likely to be victims of unprotected sex that may lead to serious social and health consequences such as HIV infection and unplanned pregnancies that would in turn lead to unsafe induced abortion. The findings suggest that health intervention programmes to prevent and control STIs, especially HIV infection, among university students should target those having the above social and behavioural factors.
